# Weight Loss Outcomes of Routine Dietetic Treatment in Primary Care

**DOI:** 10.1111/cob.70101

**Published:** 2026-08-11

**Authors:** Annemieke van de Riet, Carliene van Dronkelaar, Barbara S. van der Meij, Willemijn M. Meijer, Marian A. E. de van der Schueren, Jacqueline A. E. Langius

**Affiliations:** ^1^ Wageningen University and Research Human Nutrition and Health, Research Group Global Nutrition Wageningen the Netherlands; ^2^ Nivel (Netherlands Institute for Health Services Research) Utrecht the Netherlands; ^3^ HAN University of Applied Sciences Research Group Nutrition, Dietetics, and Lifestyle Nijmegen the Netherlands; ^4^ Faculty of Health, Nutrition and Sport, Department of Nutrition and Dietetics and Research Group Rehabilitation and Technology The Hague University of Applied Sciences The Hague the Netherlands

**Keywords:** dietitian, obesity, overweight, weight loss

## Abstract

Clinically relevant weight loss (≥ 5%) is a key treatment goal in dietetic care for patients with overweight or obesity. While clinical trials often demonstrate successful weight loss, less is known about success rates in routine dietetic practice, timing of weight loss and maintenance. This study examined when patients achieve and maintain ≥ 5% weight loss during 12 months of dietetic treatment in Dutch primary care, and how this relates to treatment intensity and patient characteristics. In this retrospective cohort study, routinely collected data from dietetic electronic health records (EHRs) was used. Follow‐up duration was 12 months. Weight change was analysed at 3, 6, 9 and 12 months. The study included 4503 adults (≥ 18 years) with overweight or obesity (BMI ≥ 25 kg/m^2^) receiving dietetic treatment between 2016 and 2020 in over 70 primary care dietetic practices in the Netherlands. The primary outcome was achieving and maintaining ≥ 5% weight loss from baseline at 3, 6, 9 and 12 months. Multivariable logistic regression assessed associations of ≥ 5% weight loss with visit frequency, visit time, sex, age, BMI and comorbidity. Overall, 33% of patients achieved ≥ 5% weight loss. Of these, 60% did so within 3 months. Each additional visit in the first 3 months was associated with threefold higher odds of success (OR = 3.0). In months 3–6, each additional visit was associated with 61% higher odds of success. Early dietetic treatment intensity is strongly associated with weight loss success. Structuring care around the early treatment phase warrants prospective evaluation in routine dietetic practice.

## Key Points

- What is already known about this subject
  ○ Dietitians play a central role in the management of overweight and obesity in primary care, but evidence on outcomes from routine dietetic practice remains limited.
  ○ Clinically relevant weight loss (≥ 5%) is associated with meaningful improvements in metabolic health and is commonly used as a treatment benchmark.
  ○ Most research on weight loss interventions has been conducted in controlled trial settings rather than in real‐world primary care dietetic practice.
- What this study adds
  ○ In routine Dutch primary care dietetic practice, one‐third of patients achieved ≥ 5% weight loss, most commonly within the first 3 months of treatment.
  ○ Treatment intensity was strongly associated with success: each additional visit in the first 3 months tripled the odds of achieving clinically relevant weight loss.
  ○ Patients who achieved early weight loss and continued treatment were more likely to maintain ≥ 5% weight loss over the first year.

## Graphical abstract

Clinically relevant weight loss (≥ 5%) is a key treatment goal in dietetic care for patients with overweight or obesity. Although clinical trials are usually highly effective in reaching this treatment goal, little is known about success rates during routine dietetic treatment, the typical timing of reaching this treatment goal, and how long it is subsequently maintained.

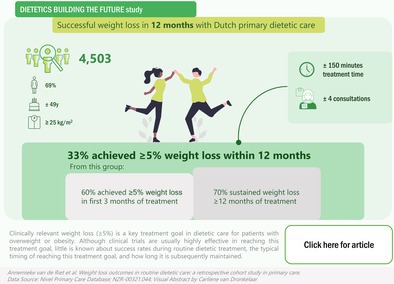

## 1 Introduction

The prevalence of overweight (body mass index [BMI] ≥ 25 kg/m^2^) and obesity (BMI ≥ 30 kg/m^2^) has risen dramatically over the past decades [1]. Globally, 43% of adults had overweight or obesity in 2022 [2]. Overweight and obesity are major risk factors for a wide range of chronic conditions, including type 2 diabetes, cardiovascular disease, certain cancers and musculoskeletal disorders, and are associated with reduced life expectancy [3, 4, 5]. In the treatment of overweight and obesity, achieving ≥ 5% weight loss has been associated with clinical improvements in health outcomes, such as better glycaemic control, lower blood pressure and lower lipid levels [6]. As a result, ≥ 5% weight loss is commonly used as a clinical benchmark for successful treatment [7, 8, 9].

In most European countries, dietitians are central in the management of overweight and obesity in primary care. They provide individualised counselling, tailored to a patient's medical, psychological and social context [10]. In 2024, 3226 primary care dietetic practices and 4890 primary care dietitians were registered in the national registry of healthcare providers in the Netherlands [11]. The Dutch health insurance system reimburses 3 h of dietetic treatment as part of the mandatory health insurance coverage [12].

While the efficacy of dietetic care in achieving weight loss has been shown in controlled trials [13, 14], the effectiveness in real‐life practice remains largely understudied. Controlled trials often involve highly motivated participants and are conducted under ideal conditions. In contrast, the real‐world setting faces challenges such as limited treatment time, variable adherence and financial or structural barriers to continuity of care [15, 16]. Achieving weight loss depends on long‐term behaviour change [17], but evidence shows that factors such as life transitions, health status, lack of accountability, limited social support and low motivation often hinder success [18, 19]. A previous observational study in Dutch dietetic primary health care found that only a quarter of patients achieved ≥ 5% weight loss with three quarter of patients having a treatment duration of ≤ 6 months [20]. Another Dutch study looking into six consecutive visits and weight loss in real life practice data found that patients with overweight and obesity had an average weight loss of 3.5%; most patients never reached the recommended threshold of ≥ 5% weight loss and did not complete the intended full year of treatment [21]. Many questions from daily practice remain unanswered. For example, at what point during treatment is clinically relevant weight loss (≥ 5%) achieved? Is this weight loss sustained over time? Furthermore, weight loss outcomes in dietetic care are shaped by a broad range of patient‐related, treatment‐related and contextual factors, including dietary approach, treatment intensity and duration and patient characteristics. Which of these factors contribute to clinically significant weight loss in Dutch primary care dietetic treatment remains insufficiently understood [20]. Most practice‐based studies report weight change only at the end of dietetic treatment, providing limited insight into the trajectory of weight loss over time. However, early weight loss has been identified as a key predictor of long‐term treatment success [22, 23]. Gaining a better understanding of when patients achieve clinically relevant weight loss could inform more personalised treatment strategies and help reduce dropout rates [12].

This study aimed to determine the proportion of patients with overweight or obesity who achieved successful (≥ 5%) weight loss within 1 year of dietetic treatment in primary care in the Netherlands. In addition, we investigated when this weight loss was first achieved, whether it was maintained during the remainder of the year, and which treatment and patient factors were associated with successful weight loss.

## 2 Methods

### 2.1 Study Design

In this retrospective cohort study, we used routinely collected data from 70 to 115 (amounts differed per year of data collection) primary care dietetic practices in the Netherlands from the Nivel Primary Care Database (Nivel‐PCD) [24]. Nivel Primary Care Database (Nivel‐PCD), maintained by Nivel, the Netherlands Institute for Health Services Research, contains longitudinal, pseudonymised, linkable extracts from electronic health record (EHR) data of participating dietitian practices among other healthcare professions in the Netherlands. These EHR contain data on the care provided (type and time) based on claim codes, health problems coded following the Dutch diagnosis coding system [25], and patient measurements of height, weight and BMI, if recorded. Ethical approval was not required as the study used anonymised secondary data from routinely collected health records.

### 2.2 Patient Selection

Patients of 18 years or older, starting dietetic treatment between 1 January 2016 and 31 December 2020, were included in the analyses. These were the most recent complete and validated data available at the start of the research project. The start of treatment was indicated with a specific claim code for ‘screening or assessment’ [26]. Patients were excluded if there was no available measurement of weight, or BMI and height (from which weight could be calculated).

Dietitians had been able to record up to four health problems per patient. The EHRs do not include the date on which health problems were recorded, making it difficult to determine their sequence of relevance in relation to the care provided. To increase the certainty that treatment was primarily aimed at weight loss, only patients whose primary health problem was coded as ‘overweight’—and who had no secondary health problems other than ‘hypertension’, ‘diabetes mellitus type II’ or ‘hypercholesterolaemia’—were included as these are obesity‐related comorbidities with also a weight loss goal.

To ensure that treatment was provided to patients with overweight or obesity, only those with a BMI of ≥ 25 kg/m^2^ at the start of treatment were included in the analyses. Furthermore, to assess weight changes from the start of treatment, only patients with at least two visits were included. Weight measurements needed to be available both at the initial visit and at least one follow‐up visit. Dutch dietary guidelines for managing overweight and obesity [27] recommend that the first follow‐up visit takes place within 2 weeks after the initial visit. To allow for possible scheduling delays, we applied a maximum interval of 5 weeks. Patients whose first follow‐up visit occurred after this period were excluded. An overview of the inclusion and exclusion criteria for the final dataset can be found in Appendix 1.

### 2.3 Outcomes

Patients were followed over time from their start visit (index date) until their last available visit or recorded weight measurement. For each available weight measurement during follow‐up, the relative change from baseline weight (i.e., weight registered at the start visit) was calculated as percentage. A weight reduction of ≥ 5% was considered successful [28]. As the time between visits varied substantially between patients, the availability and timing of weight measurements—and thus the ability to assess successful weight reduction—also varied. Therefore, follow‐up time was divided into four‐time intervals: 0–3 months (T1), 0–6 months (T2), 0–9 months (T3) and 0–12 months (T4). For each time interval, weight reduction was assessed per patient and categorised as: successful (≥ 5% reduction), not successful (< 5% reduction) or no follow‐up information available. We limited the analysis to the first 12 months of treatment because attrition increased substantially after 1 year, resulting in insufficient data for reliable evaluation beyond this period.

### 2.4 Data Analyses

Characteristics of the included patients are presented as mean ± standard deviation, number (percentage) or median (inter quartile range; IQR), as appropriate. A multivariable logistic regression was performed to assess the odds of achieving a ≥ 5% weight reduction during each time interval. BMI at the initial visit, age, sex, having one or multiple co‐morbidities (i.e., type 2 diabetes, hypercholesterolaemia and hypertension) (none vs. ≥ 1), number of visits and visit time up until the respective time interval (time dietitian spent per patient, in minutes) were added to the logistic regression as independent variables. Due to the correlation between the number of visits and visit time, an interaction term between these two variables was included in the model. Furthermore, a spaghetti plot of individual percentage weight changes during the first 12 months of treatment, with a fitted fractional polynomial line, was constructed to visualise individual weight trajectories. Kaplan–Meier analyses were performed to examine time to achieving ≥ 5% weight loss, stratified by sex. Patients were censored at their last available weight measurement. Censoring reflects loss to follow‐up; we did not model informative dropout, and the curves should be interpreted descriptively rather than causally. In order to understand how long patients continue to receive dietetic treatment after the first year and whether treatment typically ends or continues into a second year, we plotted curves for treatment duration in days and months for the period of 2 years. Data were analysed using Stata 16.1 (2020, StataCorp, Texas, USA), with statistical significance set at *p* < 0.05.

## 3 Results

The initial dataset consisted of 25 644 patients who had visited a dietitian, of whom 7835 had a registered diagnosis of high BMI (≥ 25). Most of the reduction from the initial dataset was due to patients aged under 18, patients without an available weight, height or BMI measurement and patients whose registered diagnoses did not include high BMI. After applying the remaining inclusion criteria, 4503 patients remained eligible for analysis.

The inclusion flow chart can be found in Appendix 1. Table 1 presents the baseline characteristics of the study population. The median age was 50 years, and approximately two‐thirds of the patients were female. Of the patients, 26% had overweight and 74% had obesity. The median number of dietitian visits was four (interquartile range [IQR]: 2–8), with a median treatment duration of five (IQR: 2–12) months and a median total visit time of 180 (IQR: 135–270) minutes. One‐third (33%) of the patients achieved ≥ 5% weight loss in their treatment. The median time to reach this success was 3 months (IQR: 2–5), requiring a median of four visits (IQR: 3–5). The median time spent in visits before achieving ≥ 5% weight loss was 150 min (IQR: 120–195).

**TABLE 1:** Baseline characteristics.

Patients, *n*	4503
Sex, % female	69
BMI (kg/m^2^), median (IQR)	33.1 (30.1–37.2)
BMI 25–30, %	26%
BMI ≥ 30, %	74%
Age, median (IQR)	50 (38–75)
18–44 years, %	36.7
45–64 years, %	47.0
65–84 years, %	16.1
≥ 85 years, %	0.2
Number of visits per patient, median (IQR), max	4 (2–8), 61
Treatment duration in months, median (IQR), max	5 (2–12), 64
Treatment duration < 4 months, %	44%
Treatment duration ≥ 4 months, %	56%
Treatment duration ≥ 6 months, %	43%
Treatment duration ≥ 12 months, %	24%
Treatment time in minutes, median (IQR), max	180 (135–270), 1545
Weight loss (kgs), median (IQR)	−1.28 (−3.39 to 0.00)
Proportion of patients with ≥ 5% weight loss, %	33%
Number of months in treatment after which first achievement ≥ 5% weight loss is reached, median (IQR)	3 (2–5)
Number of visits after which first achievement ≥ 5% weight loss is reached, median (IQR)	4 (3–5)
Treatment time after which first achievement ≥ 5% weight loss is reached (minutes), median (IQR)	150 (120–195)

**FIGURE 1:**
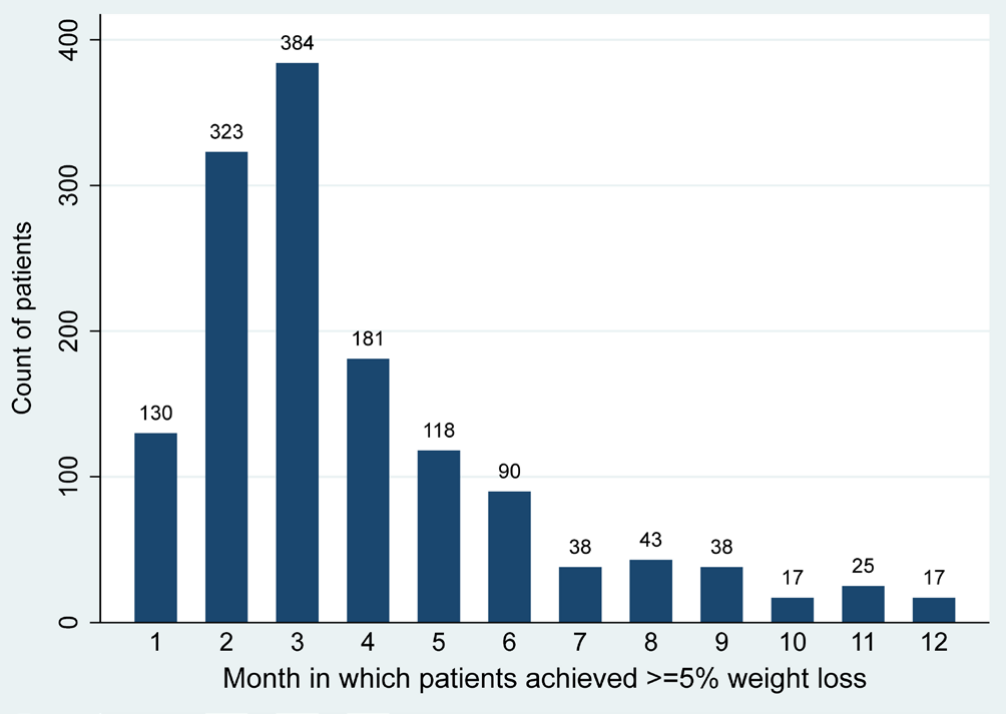
Month in which patients first achieved a ≥ 5% weight loss.

Figure 1 shows the month in which patients who had more than two consultations first achieved ≥ 5% weight loss in the first year.

**FIGURE 2:**
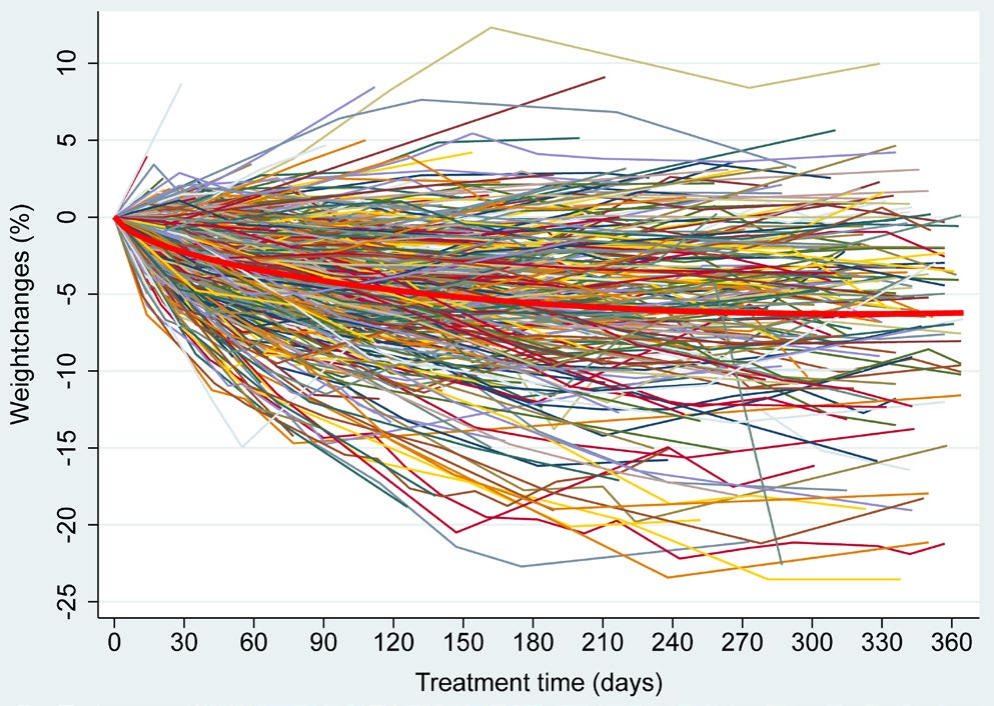
Spaghetti plot of weight changes in percentage in the first 12 months of treatment with fitted fractional polynomials line (thick, red).

Figure 2 shows individual weight trajectories during the first treatment year, with the fitted fractional polynomial line indicating an overall trend toward sustained ≥ 5% weight loss. Considerable variation was observed: some patients achieved early weight loss, others showed gradual or minimal change and many dropped out before completing the year or gained weight.

**FIGURE 3:**
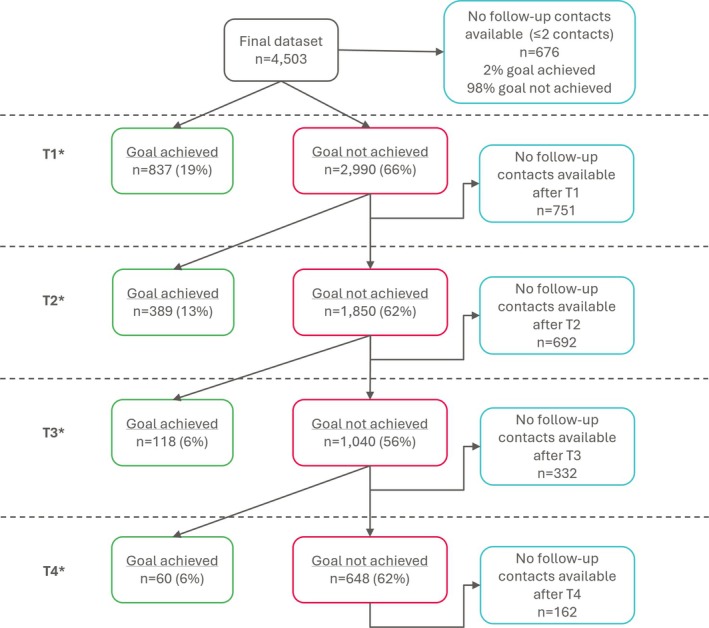
Flowchart of the patient flow over time, showing the number of patients first achieving ≥ 5% weight loss in each interval, the number not (yet) achieving it, and the number lost to follow‐up at each step (*T1: 0–3 months; T2: 0–6 months; T3: 0–9 months; T4: 9–12 months).

Figure 3 shows the flow of patients who achieved ≥ 5% weight loss and follow‐ups of those who did not achieve ≥ 5% weight loss in specified time intervals. Table 2 presents the characteristics of patients who achieved ≥ 5% weight loss for the first time within 3 months (T1), 6 months (T2), 9 months (T3) or 12 months (T4) and the odds ratios from the multivariable analysis relating patient and treatment characteristics to achieving ≥ 5% weight loss across these time points. Notably, 15% of the patients had no more than two visits with the dietitian, of whom 2% were able to achieve a weight loss of ≥ 5%.

**TABLE 2:** Descriptive characteristics and odds ratios from the multivariable analysis for patients achieving ≥ 5% weight loss versus those who did not, by time interval (T1–T4). Statistical significance shown in bold.

Total number of patients: *n* = 4503			
Treatment period	Achieving ≥ 5% weight loss	Not achieving ≥ 5% weight loss	Odds ratio (95% CI)^a^
T1 (0–3 months)^b^	*n* = 837 (19%)	*n* = 2990 (66%)	
BMI (kg/m^2^), median (IQR)	33.2 (30.2–36.2)	33.3 (30.2–37.6)	0.99 [0.97–1.00]
Age^c^ (years), median (IQR)	50 (40–60)	50 (38–60)	1.004 [0.998–1.010]
Female (%)	**67%**	**69%**	**0.74 [0.62–0.88]**
One or more comorbidity (type 2 diabetes, hypercholesterolaemia, and hypertension) (%)	**19%**	**25%**	**0.74 [0.60–0.91]**
Number of visits (*n*), median (IQR)	**3 (3–4)**	**3 (3–4)**	**3.0 [2.3–4.0]**
Total visit time minutes, median (IQR)	**150 (120–165)**	**135 (120–165)**	**1.02 [1.01–1.02]**
Visits * visit time	—		**0.996 [0.994–0.998]**

Total number of patients: *n* = 4503			
Treatment period	Goal achieved	Goal not achieved	Odds ratio (95% CI)^a^
T2 (3–6 months)^b^	*n* = 389 (13%)	*n* = 1850 (62%)	
BMI (kg/m^2^), median (IQR)	33.5 (30.4–38)	33.7 (30.5–38.1)	0.99 [0.97–1.01]
Age^c^ (years), median (IQR)	**55 (41–63)**	**50 (39–60)**	**1.02 [1.00–1.02]**
Female (%)	**64%**	**70%**	**0.72 [0.57–0.92]**
One or more comorbidity (type 2 diabetes, hypercholesterolaemia, and hypertension) (%)	24%	27%	**0.71 [0.54–0.94]**
Number of visits (*n*), median (IQR)	**5 (4–6)**	**5 (4–5)**	**1.61 [1.27–2.03]**
Visit time (min), median (IQR)	180 (150–210)	180 (150–210)	1.00 [0.998–1.01]
Visits * visit time	—	—	**0.999 [0.998–0.999]**

Total number of patients: *n* = 4503			
Treatment period	Goal achieved	Goal not achieved	Odds ratio (95% CI)^a^
T3 (6–9 months)^b^	*n* = 118 (6%)	*n* = 1040 (56%)	
BMI (kg/m^2^), median (IQR)	33.6 (29.8–38.9)	33.8 (30.7–38)	1.01 [0.98–1.04]
Age^c^ (years), median (IQR)	**53 (45–65)**	**51 (39–62)**	**1.02 [1.00–1.03]**
Female (%)	75%	70%	1.30 [0.83–2.04]
One or more comorbidity (type 2 diabetes, hypercholesterolaemia, and hypertension) (%)	35%	29%	1.12 [0.72–1.75]
Number of visits (*n*), median (IQR)	6 (5–8)	6 (5–7)	1.33 [0.98–1.81]
Total visit time (min), median (IQR)	233 (195–286)	210 (180–270)	1.01 [0.998–1.01]
Visits * visit time	—	—	0.999 [0.998–1.000]

Total number of patients: *n* = 4503			
Treatment period	Goal achieved	Goal not achieved	Odds ratio (95% CI)^a^
T4 (9–12 months)^b^	*n* = 60 (6%)	*n* = 648 (62%)	
BMI (kg/m^2^), median (IQR)	32.4 (29.9–38.3)	34 (30.8–38.3)	0.98 [0.93–1.03]
Age^c^ (years), median (IQR)	55 (42–67)	51 (40–62)	1.01 [0.99–1.03]
Female (%)	63%	71%	0.75 [0.422–1.32]
One or more comorbidity (type 2 diabetes, hypercholesterolaemia, and hypertension) (%)	37%	29%	1.29 [0.69–2.41]
Number of visits (*n*), median (IQR)	**7 (6–9)**	**7 (6–9)**	**1.80 [1.14–2.83]**
Total visit time (min), median (IQR)	255 (210–293)	255 (195–300)	1.01 [1.00–1.03]
Visits * visit time	—	—	**1.00 [1.00–1.00**]

^a^ Data is presented in means or percentage of their respective group. Odds ratios (OR) from the multivariable logistic regression for achieving ≥ 5% weight loss in the specified time period are presented. Patients without follow‐up visits can be found in Figure 4.

^b^ Each time interval represents the time since baseline: T1 = month 1, 2, 3; T2 = month 4, 5, 6; T3 = month 7, 8, 9, T4 = month 10, 11, 12.

^c^ Age is included as a continuous variable in the model.

Table 2 shows the descriptives and odds ratios of patients achieving ≥ 5% weight loss versus those who did not achieve this goal. After the first 3 months of treatment (T1), several factors were significantly associated with the odds of achieving ≥ 5% weight loss. Female patients had lower odds of reaching the goal compared to males (OR = 0.74, 95% CI: 0.62–0.88), as did those with a comorbidity (i.e., type 2 diabetes, hypercholesterolaemia and hypertension) (OR = 0.74, 95% CI: 0.60–0.91). The number of visits in this period was strongly associated with success, with each additional visit associated with threefold higher odds of achieving ≥ 5% weight loss (OR = 3.0, 95% CI: 2.3–4.0). Visit time in that specific time period was associated with a greater odds per minute of ≥ 5% weight loss (OR = 1.02, 95% CI: 1.01–1.02), although a significant interaction term between visit frequency and time (OR = 0.996, 95% CI: 0.994–0.998) showed diminishing effect when both visit frequence and time increased simultaneously. BMI and age were not significantly associated with goal achievement at T1.

Similar to 0–3 months, at 6 months (T2), being female (OR = 0.72, 95% CI: 0.57–0.92) and having a comorbidity (i.e., type 2 diabetes, hypercholesterolaemia and hypertension) (OR = 0.71, 95% CI: 0.54–0.94) were linked to lower odds of success. Age showed a small but statistically significant association (OR = 1.02, 95% CI: 1.00–1.02), indicating slightly better outcomes for older patients after 6 months. The number of visits remained significantly associated with higher odds of success, with each additional visit associated with 61% higher odds (OR = 1.61, 95% CI: 1.27–2.03), while actual visit time was not significantly associated. The interaction effect between number of visits and visit time remained statistically significant, though less pronounced (OR = 0.999, 95% CI: 0.998–0.999).

After 9 months (T3), only age showed a significant positive association, while after 12 months (T4) of treatment, only the number of visits showed a significant positive association with achieving ≥ 5% weight loss. All time periods were additionally checked for effect modification of age and sex; however, no significant interaction was found.

**FIGURE 4:**
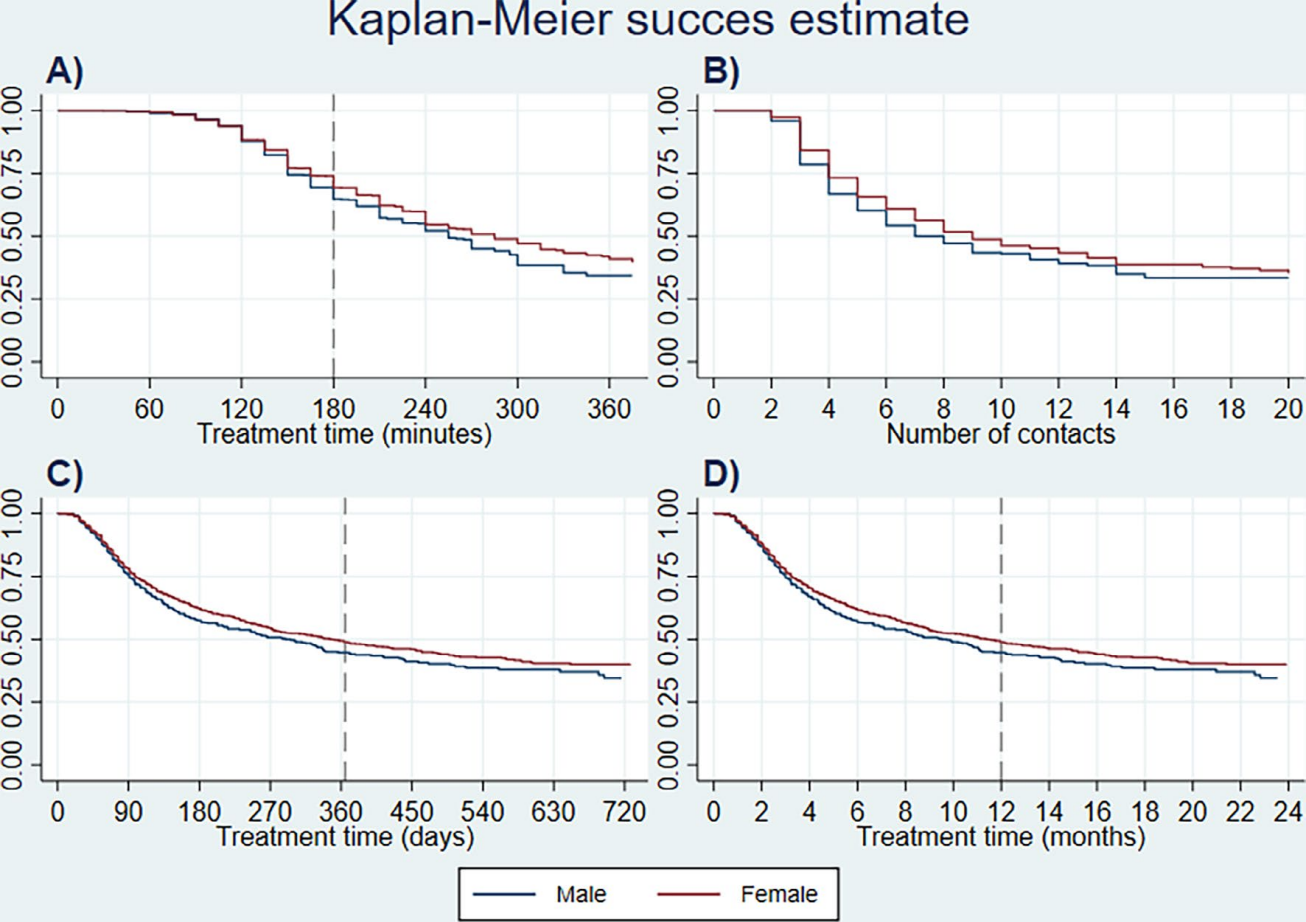
Kaplan–Meier curves for ≥ 5% weight loss as event during treatment time (A, C, D) and number of contacts (B). The vertical line in panel A represents the maximum amount of time that is covered by the Dutch health insurance (180 min) and the vertical line in panel C and D represents the 1‐year period that is analysed further in this paper.

In the Kaplan–Meier curves (Figure 4), the time to achieve ≥ 5% weight loss is shown, with each downward step of the curve representing participants reaching this outcome. The curve does not take into account that patients might have regained weight at a later moment. The top two panels display treatment intensity indicators within the first year: total treatment time in minutes (a) and number of dietetic visits (b). After approximately 180 min of treatment, only about a quarter of patients had achieved ≥ 5% weight loss. This proportion increased to nearly half of patients after around 240 min of treatment. Similarly, success rates increased with the number of visits, with the steepest gains in success rates observed up to 8–10 visits, after which the curves flattened. The bottom two panels (c, d) show treatment duration over a two‐year period, expressed in days and months. The 2‐year period was chosen to understand how long patients continue to receive dietetic treatment after the first year and whether treatment typically ends or continues into a second year. These curves indicate that most patients who eventually achieved success did so within the first year; a smaller proportion continued treatment and reached the weight loss threshold in the second year. Across all four curves, male patients reached ≥ 5% weight loss slightly earlier than female patients, although the differences were modest.

### 3.1 Maintenance of ≥ 5% Weight Loss

**FIGURE 5:**
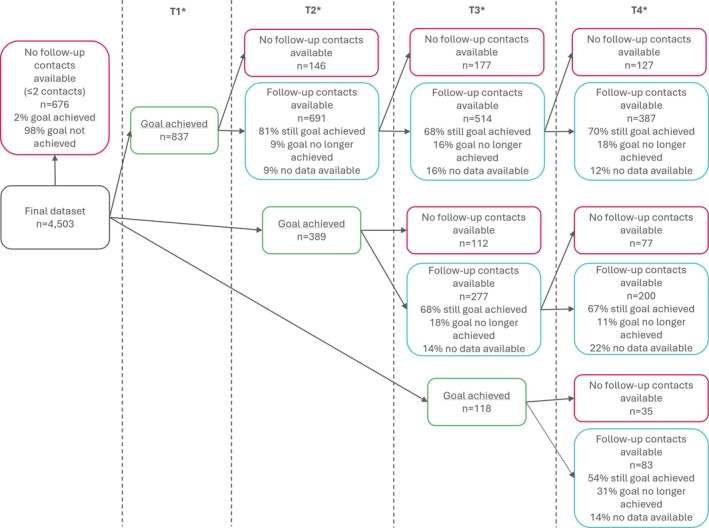
Flow of patients achieving ≥ 5% weight loss and the sustainability of this weight loss in specified time periods.

Figure 5 presents a flow diagram, illustrating those who achieved ≥ 5% weight loss after 3 months (T1), after 6 months (T2) and after 9 months (T3) of treatment, and the ability to maintain this weight loss during follow‐up visits, when available, until 12 months of treatment. As shown in the flow chart, among the patients with available data at T2, T3 and T4, the majority were able to sustain ≥ 5% weight loss. Of the 837 patients (60%) who achieved ≥ 5% weight loss within the first 3 months, 81%, 68% and 70% maintained or re‐achieved this weight loss at 6, 9 and 12 months, respectively. Among the 389 patients (28%) who first reached ≥ 5% weight loss after 6 months, 68% and 67% maintained or re‐achieved it at 9 and 12 months, respectively. Out of the 118 patients (8%) who achieved this target after 9 months of treatment (with at least three visits), 54% still had ≥ 5% weight loss at 12 months.

## 4 Discussion

In this study involving more than 4500 patients with overweight and obesity visiting a primary care dietitian in the Netherlands, we observed a clinically relevant weight loss of ≥ 5% in 33% of patients. Most of them (60%) did so within the first 3 months of treatment, while a smaller proportion reached this goal after six (28%), nine (8%) or 12 months (4%). Patients achieved ≥ 5% weight loss after a median of four visits, with a total median visit time of 150 min (2.5 h). Treatment intensity was positively associated with success: in the first 3 months, each additional visit was associated with threefold higher odds of achieving ≥ 5% weight loss (OR = 3.0), while at 6 months each additional visit was still associated with 61% higher odds (OR = 1.61). However, the interaction effect suggested that the added value of more visit time decreased when patients already had frequent sessions/visits. Female patients and those with one or more comorbidity (type 2 diabetes, hypercholesterolaemia and hypertension) were less likely to achieve the goal during the early phases, though these differences disappeared at later time points.

In our study, visit frequency emerged as a key factor associated with clinically relevant weight loss. A notable finding is that each additional visit was associated with a threefold higher likelihood of achieving ≥ 5% weight loss in the first 3 months. This is comparable to a study evaluating a weight management intervention among youth with obesity in which each additional visit per month was associated with a fivefold increase in odds of success (change in BMI ≤ 0) [29]. However, the evidence base on this association remains limited, as randomised controlled trials usually prescribe the number and timing of counselling sessions, leaving little room to examine variation in treatment intensity [26]. Moreover, the associations found in this study are observational, not causal: more motivated or responsive patients may both attend more visits and lose more weight (reverse causality). The weight loss results in our study are in line with earlier observational findings in Dutch primary care. Verberne et al. reported that approximately 25% of patients achieved ≥ 5% weight loss during dietetic treatment in Dutch primary care [20]. In our study, the slightly higher proportion of 33% was likely due to the longer follow‐up period and selection of patients with weight‐related comorbidities. In another study, Verberne et al. observed that the highest weight loss occurred between the first and second visit, and that early weight loss was associated with higher treatment adherence [21]. These findings support our observation that early success is not only common but also predictive of longer‐term outcomes. Studies have shown that greater initial weight loss improves long‐term maintenance [30, 31]. Unlike Verberne et al. [20], who found no associations with sex or age, we observed that women, as well as patients with a comorbidity (i.e., type 2 diabetes, hypercholesterolaemia and hypertension) were less likely to achieve ≥ 5% weight loss in the early stages. The lower likelihood among women was statistically significant only in the first 6 months (T1 and T2) and not thereafter, and should therefore be interpreted as an early‐phase association rather than a robust sex difference. These differences may be explained by our use of time‐stratified analysis and a clearly defined clinical threshold, rather than average weight change across the entire treatment period. The lower early success among women and patients with multimorbidity in our study is in line with other studies and may reflect more complex care needs or different treatment goals in these subgroups [32, 33, 34].

Our findings confirm that the majority of weight loss occurs within the first 6 months. This is in line with a systematic review and meta‐analysis of weight‐loss clinical trials with a minimum follow‐up of 1 year, which found that most weight loss occurs within the first 6 months of treatment, followed by a plateau [35]. The authors of the review therefore suggest an evolving programme aimed at weight loss during the first 6 months of treatment, and weight maintenance thereafter. Comparing weight loss maintenance across studies is challenging due to variations in follow‐up duration and outcome measures (e.g., absolute kilograms lost vs. relative thresholds). In our study, 54%–70% of participants who stayed in treatment and a recorded follow‐up weight, had either maintained or re‐achieved ≥ 5% weight loss threshold at different time points within the first year of treatment. It should be noted that we did not have follow‐up data from more than half of all patients after 1 year. Patients who reached ≥ 5% weight loss at the start of treatment (after 3 months) had the highest weight maintenance (70%). In comparison, Anderson et al.'s meta‐analysis reported that on average, 67% of the initial weight loss was maintained after 1 year, decreasing to 44% after 2 years and 21% after 5 years [36]. Similarly, Dansinger et al. observed that weight re‐gain often began once the intensity of treatment decreased, typically after the first year [37]. Despite different outcome metrics, these studies illustrate that substantial weight re‐gain can occur over time, calling for long‐term lifestyle changes [36, 37].

### 4.1 Strengths and Limitations

A key strength of this study is the use of a large practice‐based dataset from primary care dietetic practice, including data on treatment exposure, visit time and weight outcomes. Performing time‐based analyses allowed us to explore both when clinically relevant weight loss was achieved and whether it was maintained over time, rather than averages over the entire treatment period. These temporal insights are critical for understanding the dynamics of weight change in routine dietetic practice and may lead to more responsive and individualised treatment strategies.

However, several limitations should be noted. There was considerable variation in the frequency and duration of visits. As a result, not all patients had their weight recorded at comparable intervals, and a large proportion was lost to follow up after one or two sessions. This heterogeneity in treatment exposure and follow‐up limits the comparability between patients and time points and may lead to bias in estimating weight loss outcomes. In addition, our inclusion criteria (i.e., at least two visits with a baseline weight measurement and at least one follow‐up, with the first follow‐up within 5 weeks) were necessary to assess weight change from a comparable starting point, but they could have excluded patients who discontinued care very early or attended irregularly. The included population therefore might represent a more engaged subgroup.

Another important limitation is that the dataset did not include information on the individual treatment goals set by patients and dietitians. Although we selected only patients with comorbidities typically associated with weight loss related interventions: type 2 diabetes, hypercholesterolaemia and hypertension, it is unclear whether achieving ≥ 5% weight loss was a shared treatment goal in all cases, or whether the focus of treatment was on managing comorbidity without a clear weight loss goal. While weight loss is recommended in national dietetic guidelines for type 2 diabetes, hyperlipidaemia and hypertension [17, 38, 39], the extent to which it was prioritised in dietetic practice is unknown. Patients were censored at their last available weight measurement. Censoring reflects loss to follow‐up; we did not model informative dropout, and the curves should be interpreted descriptively rather than causally. Lack of data on reasons for treatment cessation prevented us from exploring why patients discontinued care, which is a common issue in dietetic research [37, 40]. In addition, we had insufficient follow‐up data to investigate weight loss maintenance after 1 year, while other studies have shown that weight regain often begins after the first year when dietary support was gradually reduced [41, 42]. Finally, while this dataset was used to evaluate a clinically significant weight loss of ≥ 5%, it is important to acknowledge that we lacked data on important patient data and factors that have been suggested to influence treatment engagement and outcomes, such as socioeconomic status and motivation, psychiatric comorbidity, referral pathway, dietitian characteristics and the dietary or behavioural approach used, as well as information on the patient‐dietitian relationship [15, 43]. Residual confounding by these unmeasured factors may affect the reported associations, and subgroup findings should be interpreted with caution. Although ≥ 5% weight loss is a clinically meaningful and widely used threshold, dichotomising weight change does not capture the magnitude of loss or its stability over time. Besides weight loss, other important measures for improved health outcomes in patients with obesity are reduction of waist circumference and improved quality of life [8], which we did not have data on. Our data also lacked patient‐reported outcome measures (PROMs) and patient‐reported experience measures (PREMs), which limits a comprehensive understanding of treatment success [44]. Furthermore, a substantial proportion of patients discontinued treatment after only one or two sessions and were lost to follow‐up. This group remains poorly understood, yet their early dropout is likely to undermine both clinical outcomes and the cost‐effectiveness of dietetic care. Finally, the dataset covers 2016–2020, which may not fully reflect more recent developments in primary care dietetics, such as the increased use of digital or blended care following the COVID‐19 pandemic or the introduction of GLP‐1 agonists for the treatment of obesity [8]. The results of this study are likely generalisable to routine primary care dietetic practice in the Netherlands, as the dataset reflects real‐world care across a representative group of dietetic practices. Generalisability to other health care systems should be interpreted cautiously due to differences in organisation and reimbursement of dietetic care.

### 4.2 Practical Implications and Future Research

We found that most patients who lose ≥ 5% of their weight do so in the first 3 months of treatment, and that every extra visit was associated with a threefold higher chance of success in that period. This has important implications for dietetic practice. It suggests that the early phase of treatment is critical for initiating change and achieving measurable outcomes and that the number of visits is important for successful weight loss. Dietitians may take this into account when spreading the 3 h of reimbursed treatment per year. However, as these findings are observational, it cannot be concluded that increasing the number of early visits in itself improves outcomes; this should be confirmed in prospective studies.

More research is needed into long‐term outcomes after dietetic treatment and into changes in body composition (including muscle quantity and muscle quality) with weight loss. Such insights could inform adaptations aimed at improving treatment retention and treatment effectiveness. Furthermore, standard registration on PROMs, including quality of life, and PREMs can help to also measure the experience of dietetic treatment.

## 5 Conclusion

This practice‐based study shows that 33% of patients with overweight or obesity in Dutch primary dietetic care achieved clinically relevant weight loss, with 60% of the patients reaching this threshold within the first 3 months. Patients typically achieved success after only a few visits, and those who lost weight early and continued treatment were most likely to maintain their results throughout the year. A key finding was that visit was strongly associated with success: in the first 3 months, each additional visit was associated with threefold higher odds of achieving ≥ 5% weight loss. In Months 3–6, each additional visit was associated with 61% higher odds of success. These results underline that the early phase of treatment is important for initiating weight change, and that the number of visits plays a central role in achieving and sustaining clinically meaningful outcomes. Dietitians may consider more intense support in the initial treatment months by increasing visit frequency. However, as these findings are observational, it cannot be concluded that increasing the number of early visits will in itself improve outcomes; this should be confirmed in prospective studies. Future research should focus on understanding differences in treatment response and on improving engagement and long‐term weight maintenance.

## Author Contributions

J.A.E.L., B.S.v.d.M. and M.A.E.d.v.d.S. designed the study, were involved in planning and supervised the work. A.v.d.R. and C.v.D. processed the data. C.v.D. performed the analyses. W.M.M. supervised the data analyses. All authors were involved in the interpretation of the data. A.v.d.R. and C.v.D. drafted the manuscript and designed the figures and tables. All authors provided critical feedback and helped shape the research, analysis and manuscript and contributed to the final version of the manuscript.

## Funding

This work was supported by the ZonMw under the programme Allied Health Care 2019–2022 (10270032021001). ZonMw is a Dutch research funding agency advancing health and healthcare innovations and was not involved in this study.

## Conflicts of Interest

The authors declare no conflicts of interest.

## Supporting information


**Appendix 1** Inclusion flow chart.
**Figure A1**. Flowchart of patients' inclusion in the final dataset.
**Table STROBE:‐nut**: An extension of the STROBE statement for nutritional epidemiology.

## Data Availability

In the present study, data from the Nivel Primary Care Database (Nivel‐PCD) have been used. However, access to data in Nivel‐PCD is subject to Nivel‐PCD governance codes. Requests for access to data can be directed at gegevensaanvragen@nivel.nl. Restrictions involve establishing a data sharing agreement and approval by the appropriate Nivel‐PCD governance bodies (privacy committee and steering committee). Data were derived from routine electronic health record systems of dietetic practices participating in Nivel Primary Care Database (Nivel‐PCD). The data request is registered under the number: NZR‐00321.044. The use of electronic health records for research purposes is allowed under certain conditions. When these conditions are fulfilled, neither obtaining informed consent from patients nor approval by a medical ethics committee is obligatory for this type of observational studies containing no directly identifiable data (art. 24 GDPR implementation Act jo art. 9.2 sub j GDPR).
